# Ibrutinib versus rituximab in relapsed or refractory chronic lymphocytic leukemia or small lymphocytic lymphoma: a randomized, open‐label phase 3 study

**DOI:** 10.1002/cam4.1337

**Published:** 2018-03-13

**Authors:** Xiaojun Huang, Lugui Qiu, Jie Jin, Daobin Zhou, Xiequn Chen, Ming Hou, Jianda Hu, Yu Hu, Xiaoyan Ke, Junmin Li, Yingmin Liang, Ting Liu, Yue Lv, Hanyun Ren, Aining Sun, Jianmin Wang, Chunting Zhao, Mariya Salman, Steven Sun, Angela Howes, Jingzhao Wang, Peng Wu, Jianyong Li

**Affiliations:** ^1^ Peking University People's Hospital Beijing China; ^2^ Institute of Hematology & Blood Diseases Hospital Chinese Academy of Medical Sciences Tianjin China; ^3^ The First Affiliated Hospital Zhejiang University College of Medicine Zhejiang China; ^4^ Peking Union Medical College Hospital Beijing China; ^5^ Xijing Hospital Shanxi China; ^6^ Qilu Hospital of Shandong University Jinan China; ^7^ Fujian Medical University Union Hospital Fuzhou China; ^8^ Wuhan Union Hospital Wuhan China; ^9^ Peking University Third Hospital Beijing China; ^10^ Ruijin Hospital Shanghai China; ^11^ Tangdu Hospital Shanxi China; ^12^ West China Hospital Chengdu China; ^13^ Sun Yat‐Sen University Cancer Center Guangzhou China; ^14^ The First Affiliated Hospital of Soochow University Suzhou China; ^15^ Changhai Hospital The Second Military Medical University Shanghai China; ^16^ The Affiliated Hospital of Qingdao University Medical College Qingdao China; ^17^ Janssen Research & Development Raritan New Jersey; ^18^ Janssen Research & Development High Wycombe UK; ^19^ Janssen China Research & Development Center Beijing China; ^20^ Jiangsu Province Hospital Nanjing China

**Keywords:** Asia‐Pacific, chronic lymphocytic leukemia, ibrutinib, rituximab, small lymphocytic lymphoma

## Abstract

In the Asia‐Pacific region, treatment options are limited for patients with relapsed/refractory chronic lymphocytic leukemia (CLL)/small lymphocytic lymphoma (SLL). Rituximab is widely used in this setting when purine analog‐based therapies are not appropriate. We evaluated the efficacy and safety of ibrutinib compared with rituximab in a randomized, open‐label phase 3 study in predominantly Asian patients with relapsed/refractory CLL/SLL. Patients (*N* = 160) were randomly assigned 2:1 to receive 420 mg ibrutinib (*n* = 106) until disease progression (PD) or unacceptable toxicity or up to six cycles of rituximab (*n* = 54). The primary endpoint was investigator‐assessed progression‐free survival (PFS); key secondary endpoints were overall response rate (ORR), overall survival (OS), and safety. Rituximab‐treated patients could crossover to receive ibrutinib after confirmed PD. At data cutoff, median treatment duration was 16.4 months for ibrutinib and 4.6 months for rituximab. Ibrutinib significantly improved PFS (hazard ratio [HR] = 0.180, 95% confidence interval [CI]: 0.105–0.308). ORR was significantly higher (*P *<* *0.0001) with ibrutinib (53.8%) than with rituximab (7.4%). At a median follow‐up of 17.8 months, ibrutinib improved OS compared with rituximab (HR = 0.446; 95% CI: 0.221–0.900; *P *=* *0.0206). Overall incidence of adverse events (AEs) was similar between treatments and was not exposure‐adjusted. With ibrutinib, most common AEs were diarrhea and platelet count decreased; with rituximab, most common AEs were neutrophil count decreased and platelet count decreased. Grade ≥3 AEs were reported in 82.7% of ibrutinib‐treated patients and 59.6% of rituximab‐treated patients. Ibrutinib improved PFS, ORR, and OS compared with rituximab and displayed a manageable safety profile in Asian patients with relapsed/refractory CLL/SLL.

## Introduction

Chronic lymphocytic leukemia (CLL) is the most common adult leukemia in the Western world with an estimated incidence of 4.2 cases per 100,000/year 1. The incidence rate in Asia is lower at <1 case per 100,000/year 2, 3, 4, 5. It is unknown whether this difference is related to environmental or genetic factors, but patients of Asian descent who live in Western countries also show a lower incidence of CLL 6, 7, suggesting the importance of genetics in disease risk. The frequency of chromosomal aberrations and mutations associated with CLL is similar between Asian and Western patients 8, 9, 10, 11. A slightly higher prevalence of mutated immunoglobulin heavy chain variable region (IGVH) has been associated with Chinese patients 8, 12, and studies in Japanese and Korean patients show an increased frequency of CLL with atypical immunophenotypes 13, 14. Although there may be differences in genetic and pathological features of CLL, the efficacy of CLL treatments in Asian and Western patients has been comparable 15, 16, 17.

Advances in targeted therapies for CLL/small lymphocytic lymphoma (SLL) have led to remarkable improvements in clinical outcomes. However, in the Asia‐Pacific region, available treatment options are limited, especially in the relapsed/refractory (R/R) setting. The standard treatment for CLL/SLL is chemoimmunotherapy in young or fit patients 1, 18, but elderly patients and patients with comorbidities are often unable to tolerate the aggressive regimens and experience poor clinical outcomes 19. Moreover, patients with genetic aberrations associated with high‐risk CLL/SLL, including the 17p deletion (del17p), respond poorly to standard chemoimmunotherapy 20, 21. For these difficult‐to‐treat patients who are not appropriate candidates for traditional chemotherapy approaches, treatment options in China were limited to rituximab and lenalidomide/thalidomide.

Ibrutinib is a potent covalent inhibitor of Bruton's tyrosine kinase, a key component of B‐cell signaling that plays an important role in B‐cell development, survival, and function 22. Ibrutinib is approved in the United States and Europe for the treatment of mantle cell lymphoma (in patients who have received ≥1 prior therapy), CLL/SLL, Waldenström's macroglobulinemia, and marginal zone lymphoma (US only) 23, 24. Recently, ibrutinib was approved in China for the treatment of CLL/SLL and mantle cell lymphoma. Based on the unprecedented improvements in progression‐free survival (PFS) and overall survival (OS) observed with single‐agent ibrutinib in R/R CLL, the National Comprehensive Cancer Network (NCCN) recommended single‐agent ibrutinib as a category 1 recommendation in the R/R setting regardless of del17p status 18.

At the time this study was initiated, the efficacy of single‐agent ibrutinib had only been demonstrated in a phase 2 study in the United States, which largely enrolled white patients 25. We conducted a study to determine whether ibrutinib could demonstrate comparable safety and efficacy in a predominantly Asian population of patients. At the time this study was conducted, ibrutinib was not approved in any of the countries that participated in the trial. This study also overlapped with a global study which compared ibrutinib with ofatumumab in a similar R/R CLL/SLL population 26. Because of the lack of standard treatment and the limited treatment options for R/R CLL/SLL in the Asia‐Pacific region, rituximab monotherapy is widely used in China for treatment of patients with R/R CLL/SLL for whom purine analog‐based therapies are not suitable.

Rituximab is an anti‐CD20 chimeric monoclonal antibody capable of inducing antibody‐dependent cell‐mediated cytotoxicity and complement‐dependent cytotoxicity 27. Although rituximab monotherapy is not considered a preferred treatment option for first‐line or R/R CLL/SLL, it was a reasonable choice as a comparator based on the limited treatment options available at the time this study was initiated. Rituximab is listed as a treatment option for patients unable to tolerate purine analog therapy in the NCCN treatment guidelines 18 and has been the comparator in another global study of a B‐cell signaling inhibitor 28. In other global regions, rituximab monotherapy is a commonly prescribed treatment option in the R/R setting 29, 30, 31, 32, 33. A real‐world analysis using the 2007–2013 SEER‐Medicare database in the United States showed that rituximab monotherapy was used as second‐line therapy in 30.5% of patients 29. Here, we report our findings from a randomized, single‐agent, open‐label, multicenter, phase 3 study comparing the efficacy and safety of ibrutinib with rituximab in a predominantly Asian population of patients with R/R CLL/SLL.

## Materials and Methods

### Patients

This randomized, open‐label, multicenter, phase 3 study was conducted at 29 sites in China, Australia, Taiwan, and Malaysia (http://www.ClinicalTrials.gov; NCT01973387). Eligible patients were ≥18 years with a diagnosis of active CLL/SLL that required treatment according to the International Workshop on Chronic Lymphocytic Leukemia (IWCLL) 2008 criteria 34, received at least 1 prior therapy for CLL/SLL, and were not considered appropriate candidates for purine analog‐based therapy. Additional inclusion and exclusion criteria are described in the Appendix S1.

### Study design

Patients were randomly assigned 2:1 to receive 420 mg oral ibrutinib once daily or intravenous rituximab. Randomization was stratified by purine analog refractory status (yes or no) and the presence of del17p (yes or no). Patients were assigned to a treatment group between 26 December 2013 and 15 September 2015 using an interactive web response system. Rituximab was administered at 375 mg/m^2^ on day 1 and 500 mg/m^2^ on day 15 of cycle 1; 500 mg/m^2^ on days 1 and 15 for cycle 2; and 500 mg/m^2^ on day 1 of cycles 3–6. Rituximab dosing was based on the dosing regimen used in fludarabine, cyclophosphamide, and rituximab therapy and similar to the dosing schedule used in a previously reported study of single‐agent rituximab in patients with relapsed CLL 28.

The study consisted of three phases: a screening phase, treatment phase, and follow‐up phase (Fig. S1). Patients were screened for eligibility up to 28 days prior to randomization. The treatment phase extended from randomization until study drug discontinuation. Patients received daily ibrutinib until PD or unacceptable toxicity. Patients received up to six cycles of rituximab until disease progression or unacceptable toxicity, whichever occurred first. The follow‐up phase consisted of two phases: the post‐treatment phase and a postdisease progression phase. The post‐treatment phase extended from the discontinuation of treatment (for reasons other than PD) until PD, at which point the postdisease progression phase began. During the postdisease progression phase, subsequent anticancer therapies and survival status were recorded until death, loss to follow‐up, withdrawal of consent, or study closure.

The primary endpoint was investigator‐assessed PFS, defined as the time from randomization until PD per IWCLL 2008 criteria 34 or death, whichever occurred first. The criteria for PD are described in the Appendix S1. Key secondary endpoints were ORR, OS, pharmacokinetics (PK) in Chinese patients, and safety.

Assessments for response and progression for both treatment arms were conducted until PD in accordance with the IWCLL 2008 criteria; treatment‐related lymphocytosis was not considered as PD per IWCLL guidelines 35. The criteria for response categories are described in the Appendix S1 and in Table S1. Safety assessments included adverse events (AEs), physical examination, laboratory tests, and vital signs.

Following an amendment to the protocol, eligible patients in the rituximab arm who had investigator‐assessed PD were permitted to cross over to receive ibrutinib after PD was confirmed by a central independent physician (Fig. S2). The study was approved by the institutional review board or independent ethics committee at each site and was conducted in accordance with the principles of the Declaration of Helsinki and the International Conference on Harmonization Good Clinical Practice guidelines. All patients provided written informed consent.

### Statistical methods

Approximately 150 patients were planned to be randomized to observe 90 PFS events. The study was designed to detect a hazard ratio (HR) of 0.54 for the ibrutinib arm relative to the rituximab arm with 80% power at a 1‐sided significance level of 0.025. An interim analysis using O'Brien‐Fleming boundary for superiority was planned after approximately 45 PFS events. The stopping boundary was implemented by Lan‐DeMets alpha‐spending function resembling the O'Brien‐Fleming boundary using East software, version 6.3 (Cytel, Cambridge, MA, USA) to control the overall 1‐sided Type I error of 0.025 on the PFS endpoint.

The intent‐to‐treat (ITT) population included all patients randomized into the study and analyzed according to assigned treatment group, regardless of the actual treatment received. The safety population included all patients who received at least one dose of study drug and was analyzed as actual treatment received.

The primary efficacy analysis of PFS in the ITT population was compared using a stratified log‐rank test based on the stratification factors. Distribution of PFS was summarized using median and corresponding 95% confidence interval (CI) based on Kaplan–Meier estimates. The estimate of the HR and its corresponding 95% CI were calculated using a Cox proportional hazards model stratified by stratification factors. A preplanned subgroup analysis of PFS based on prognostic variables was conducted. An ad hoc analysis of PFS was conducted using a multivariate Cox regression model with all key prognostic factors as covariates: treatment, age, sex, Rai stage at screening, baseline Eastern Cooperative Oncology Group score, prior lines of therapy, chromosome 11q deletion (del11q), bulky disease, refractory to purine analog therapy, and del17p.

Overall response rate was estimated according to the proportion of responders (complete response [CR] + partial response [PR]) based on the best overall response and summarized by treatment. All responses were confirmed responses, defined as maintained for at least 2 months without transfusion or growth factors. ORR was compared using the Cochran–Mantel–Haenszel chi‐square test. Lymph node response rate, which was defined as the proportion of patients who had ≥50% reduction in lymphadenopathy or with all measured lymph nodes normalized, was included as a sensitivity analysis for ORR.

Overall survival was estimated with deaths due to any cause in the study considered as events. Distribution of OS was summarized for each treatment arm using median and its corresponding 95% CI based on Kaplan–Meier estimates. The HR estimate and its corresponding 95% CI were calculated using a Cox proportional hazards model stratified by the stratification factors.

Adverse events were summarized, and the severity of AEs was graded according to the adult National Cancer Institute Common Terminology Criteria for Adverse Events, version 4.03 or protocol definition. Hematologic AEs were assessed by the IWCLL 2008 criteria 34.

## Results

### Patients

In total, 160 eligible patients were randomized; 106 patients to the ibrutinib arm and 54 patients to the rituximab arm (Fig. S3). More men (70.6%) were enrolled in the study than women (29.4%), and 85% of patients were Chinese (Table 1). The majority of patients were elderly, with a median age of 66 years (range, 21–87 years). A large proportion of patients had advanced‐stage CLL with high‐risk clinical features including bulky disease (43.8%), del11q (21.3%), del17p (22.5%), and unmutated IGVH (61.3%; Table 1). Patient demographics and characteristics were generally comparable between treatment groups. Prior monoclonal antibody therapy was received by 36.9% of patients; prior rituximab treatment was received by 32.1% of the ibrutinib arm and 44.4% of the rituximab arm. Patients with ≥3 prior CLL/SLL therapies, Rai stage 1, prior purine analog therapy, bulky disease, hemoglobin ≤11 g/dL, and absolute neutrophil count ≤1500/*μ*L were also more prevalent in the rituximab arm (Table 1).

**Table 1:** Demographics and baseline disease characteristics (ITT population)

	Ibrutinib (*n* = 106)	Rituximab (*n* = 54)	Total (*N* = 160)
Age			
Category, *n* (%)			
<65	52 (49.1)	23 (42.6)	75 (46.9)
≥65 to 69	22 (20.8)	12 (22.2)	34 (21.3)
≥70	32 (30.2)	19 (35.2)	51 (31.9)
Mean (SD)	63.6 (10.4)	63.6 (13.0)	63.6 (11.3)
Median	65	67	66
Range	(39, 87)	(21, 86)	(21, 87)
Sex, *n* (%)			
Female	29 (27.4)	18 (33.3)	47 (29.4)
Male	77 (72.6)	36 (66.7)	113 (70.6)
Race, *n* (%)			
Chinese	91 (85.8)	45 (83.3)	136 (85.0)
White	14 (13.2)	8 (14.8)	22 (13.8)
Asian, not Chinese	1 (0.9)	0	1 (0.6)
Other	0	1 (1.9)	1 (0.6)
Initial diagnosis to randomization (months)			
Mean (SD)	54.7 (57.8)	64.9 (58.7)	58.1 (58.1)
Median	40.1	45.9	41.1
Range	(0.0, 405.4)	(3.8, 283.5)	(0.0, 405.4)
Initial diagnosis, *n* (%)			
CLL	100 (94.3)	51 (94.4)	151 (94.4)
SLL	6 (5.7)	3 (5.6)	9 (5.6)
Baseline Rai stage (CLL only), *n* (%)			
*N*	99	51	150
0	0	0	0
I	9 (9.1)	11 (21.6)	20 (13.3)
II	11 (11.1)	3 (5.9)	14 (9.3)
III	18 (18.2)	9 (17.6)	27 (18.0)
IV	61 (61.6)	28 (54.9)	89 (59.3)
Baseline Binet stage (CLL only), *n* (%)			
*N*	100	51	151
A	2 (2.0)	4 (7.8)	6 (4.0)
B	25 (25.0)	10 (19.6)	35 (23.2)
C	73 (73.0)	37 (72.5)	110 (72.8)
Prior purine analog therapy, *n* (%)
Yes	69 (65.1)	42 (77.8)	111 (69.4)
Failed to respond	28 (26.4)	12 (22.2)	40 (25.0)
Relapse <6 months	9 (8.5)	7 (13.0)	16 (10.0)
Relapse ≥6 to <12 months	5 (4.7)	6 (11.1)	11 (6.9)
Relapse ≥12 to <24 months	13 (12.3)	6 (11.1)	19 (11.9)
Relapse ≥24 months	10 (9.4)	9 (16.7)	19 (11.9)
Not evaluable/unknown	4 (3.8)	2 (3.7)	6 (3.8)
No	37 (34.9)	12 (22.2)	49 (30.6)
Number of prior CLL/SLL therapies			
*N*	105	54	159
Category, *n* (%)			
1	55 (52.4)	23 (42.6)	78 (49.1)
2	24 (22.9)	11 (20.4)	35 (22.0)
≥3	26 (24.8)	20 (37.0)	46 (28.9)
Mean (SD)	2.0 (1.7)	2.2 (1.4)	2.1 (1.6)
Prior rituximab treatment, *n* (%)	34 (32.1)	24 (44.4)	58 (36.3)
ECOG performance status, *n* (%)			
0	54 (50.9)	23 (42.6)	77 (48.1)
1	52 (49.1)	31 (57.4)	83 (51.9)
Bulky disease, *n* (%)			
Yes (≥5 cm)	42 (39.6)	28 (51.9)	70 (43.8)
No (<5 cm)	64 (60.4)	26 (48.1)	90 (56.3)
Chromosome 11q deletion, *n* (%)			
Yes	22 (20.8)	12 (22.2)	34 (21.3)
No	84 (79.2)	42 (77.8)	126 (78.8)
Chromosome 17p deletion, *n* (%)			
Yes	23 (21.7)	13 (24.1)	36 (22.5)
No	83 (78.3)	41 (75.9)	124 (77.5)
IGVH status, *n* (%)			
Mutated	33 (31.1)	16 (29.6)	49 (30.6)
Unmutated	63 (59.4)	35 (64.8)	98 (61.3)
Unevaluable	10 (9.4)	3 (5.6)	13 (8.1)
Cytopenia at baseline^a^, *n* (%)			
Yes	82 (77.4)	43 (79.6)	125 (78.1)
Platelet count ≤100,000/*μ*L	69 (65.1)	32 (59.3)	101 (63.1)
Hgb ≤11 g/dL	49 (46.2)	32 (59.3)	81 (50.6)
ANC ≤1500/*μ*L	15 (14.2)	18 (33.3)	33 (20.6)
No	24 (22.6)	11 (20.4)	35 (21.9)

ANC, absolute neutrophil count; CLL, chronic lymphocytic leukemia; ECOG, Eastern Cooperative Oncology Group; Hgb, hemoglobin; IGVH, immunoglobulin heavy chain variable region; ITT, intent‐to‐treat; SD, standard deviation; SLL, small lymphocytic lymphoma.

a Cytopenia defined as platelet count ≤100,000/*μ*L, Hgb ≤11 g/dL, or ANC ≤1500/*μ*L.

Percentages were calculated with the number of patients in the ITT analysis set in each treatment group with nonmissing values for that parameter as the denominator.

In January 2016, a planned interim analysis was conducted after 48 PFS events were observed; the clinical cutoff date was 1 December 2015. The independent data monitoring committee (DMC) reviewed the unblinded safety and efficacy data and confirmed that the prespecified statistical boundary for early efficacy stopping was crossed. The DMC recommended early analysis and stopping of the study for efficacy. We conducted an updated analysis that included follow‐up data with a cutoff date of 14 April 2016. The data from the updated analysis with longer follow‐up will be presented here; data from the interim analysis and updated analysis were consistent (Table S2).

At the data cutoff date of 14 April 2016, 36 patients from the rituximab arm completed all six cycles of treatment. Early treatment discontinuation was reported for 32 (30.2%) ibrutinib‐treated patients and 16 (29.6%) rituximab‐treated patients (Fig. S3). The reasons for treatment discontinuation in the ibrutinib arm were AEs (12.3%), PD/relapse (9.4%), withdrawal of consent (5.7%), and death (2.8%); the reasons for treatment discontinuation in the rituximab arm were PD/relapse (9.3%), AEs (7.4%), withdrawal of consent (7.4%), and death (5.6%). Twenty (37.0%) patients in the rituximab arm crossed over to receive next line ibrutinib treatment after confirmed PD. The median duration of exposure (measured from the first date of study drug dose to the last date of study drug dose) was 16.4 months for ibrutinib and 4.6 months for rituximab. Seventy‐two (67.9%) patients in the ibrutinib arm and no patients in the rituximab arm remained on treatment.

### Efficacy

**Figure 1:**
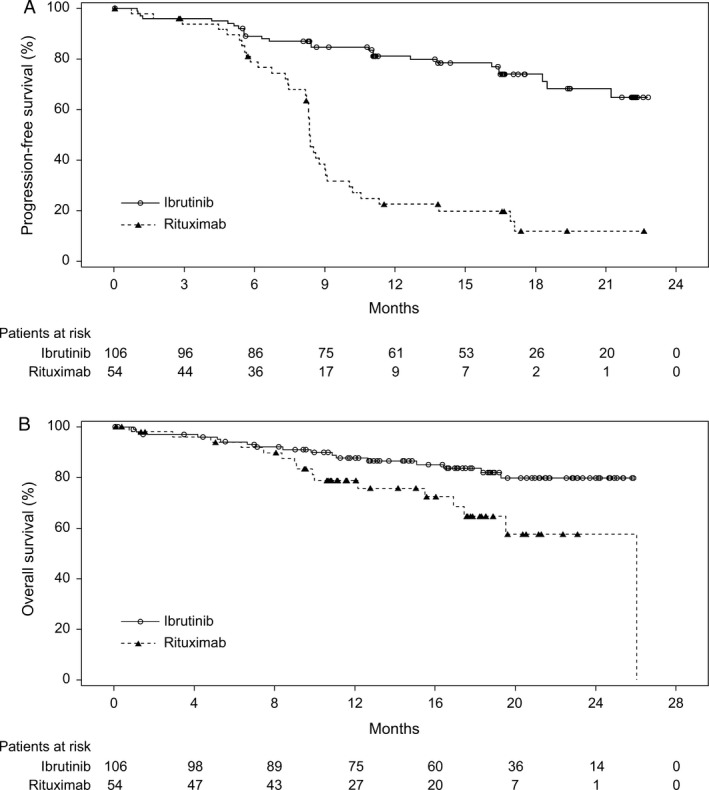
(A) Kaplan–Meier Curves for Progression‐Free Survival (ITT Population). The median progression‐free survival (PFS) was not reached in the ibrutinib arm. The median PFS for the rituximab arm was 8.34 months (95% confidence interval: 8.21–9.03 months). (B) Kaplan–Meier Curves for Overall Survival (ITT Population). With a median follow‐up of 17.84 months, overall survival was significantly improved in the ibrutinib arm compared with the rituximab arm.

In the updated analysis, 64 PFS events were reported (26 [24.5%] in the ibrutinib arm and 38 [70.4%] in the rituximab arm). PFS was significantly improved for patients in the ibrutinib arm compared with the rituximab arm (HR = 0.180, 95% CI: 0.105–0.308; *P *<* *0.0001). The median PFS was not reached in the ibrutinib arm; median PFS for the rituximab arm was 8.3 months (range, 0–22.6 months). At the 18‐month landmark, the estimated PFS rate in the ibrutinib arm was 74.0%, and in the rituximab arm, it was 11.9% (Fig. 1A).

**Figure 2:**
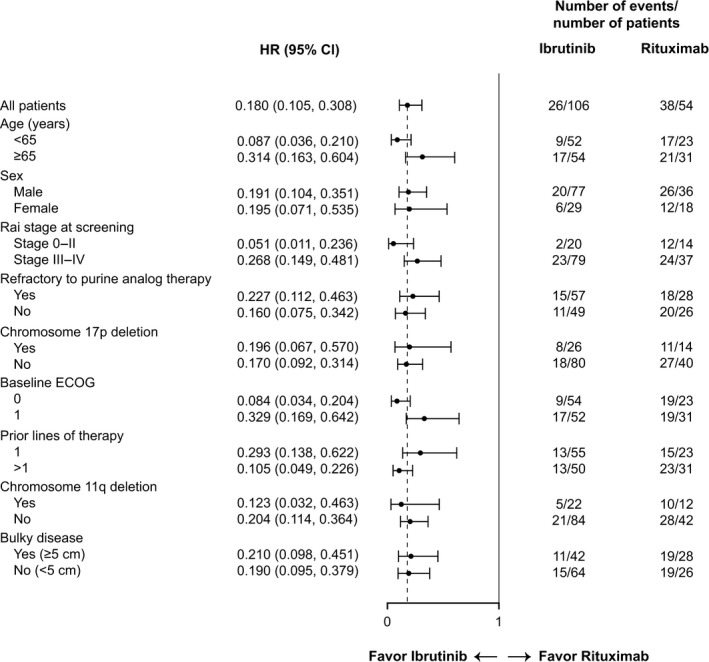
Forest Plot of Progression‐Free Survival by Subgroup. Hazard ratios (HRs) <1 favor ibrutinib, and HRs greater than 1 favor rituximab. The HR for each subgroup is represented by a black circle, and 95% confidence intervals (CI) are denoted by the brackets. The dotted line represents the HR (0.18) for all patients. ECOG, Eastern Cooperative Oncology Group.

The improvement in PFS in the ibrutinib arm compared with the rituximab arm was observed in all subgroups examined (Fig. 2). Results from the subgroup analysis were consistent with those observed for the overall population. A multivariate Cox regression analysis of PFS showed that treatment effects remained robust even after controlling for key prognostic factors (Table 2).

**Table 2:** Multivariate cox regression analysis of PFS (ITT population)

Model	HR (95% CI)	*P*‐value
Treatment group: ibrutinib vs. rituximab	0.178 (0.101–0.316)	<0.0001
Age: ≥65 vs. <65	0.706 (0.379–1.315)	0.2724
Sex: male vs. female	1.056 (0.589–1.893)	0.8548
Rai stage at screening: stage 0‐II vs. stage III‐IV	0.746 (0.393–1.416)	0.3706
Baseline ECOG: 1 vs. 0	1.305 (0.755–2.258)	0.3405
Prior lines of therapy: >1 vs. 1	1.342 (0.775–2.324)	0.2943
Chromosome 11q deletion: yes vs. no	1.019 (0.538–1.929)	0.9540
Bulky disease: yes vs. no	1.309 (0.779–2.199)	0.3089
Refractory to purine analog therapy: yes vs. no	0.847 (0.467–1.536)	0.5839
Chromosome 17p deletion: yes vs. no	1.012 (0.555–1.844)	0.9693

CI, confidence interval; ITT, intent‐to‐treat; ECOG, Eastern Cooperative Oncology Group; HR, hazard ratio; PFS, progression‐free survival.

Overall response rate (CR+PR) was significantly higher for the ibrutinib arm (53.8%) than for the rituximab arm (7.4%; Table 3). The response rate ratio was 7.32 (95% CI: 2.79–19.18; *P *<* *0.0001), indicating a significant increase in the chance of response with ibrutinib. CR was achieved in four (3.8%) patients in the ibrutinib arm; no CRs were reported in the rituximab arm. The ORR including PR with lymphocytosis (PRL; CR+PR+PRL) was also significantly higher for the ibrutinib arm (67.9%) than for the rituximab arm (7.4%), with a rate ratio of 9.24 (95% CI: 3.54–24.06*; P *<* *0.0001).

**Table 3:** Summary of overall response rate (ITT population)

	Ibrutinib (*n* = 106)	Rituximab (*n* = 54)	Ibrutinib vs. rituximab
Overall response rate (CR, CRi, nPR, PR)	57 (53.8)	4 (7.4)	
Rate ratio (95% CI)^a^			7.32 (2.79–19.18)
*P*‐value^a^			<0.0001
Overall response rate including PRL (CR, CRi, nPR, PR, PRL)	72 (67.9)	4 (7.4)	
Rate ratio (95% CI)^a^			9.24 (3.54–24.06)
*P*‐value^a^			<0.0001
Best overall response			
Complete response	4 (3.8)	0	
Complete response with incomplete marrow recovery	0	0	
Nodular partial response	0	0	
Partial response	53 (50.0)	4 (7.4)	
Partial response with lymphocytosis	15 (14.2)	0	
Stable disease	23 (21.7)	43 (79.6)	
Progressive disease	1 (0.9)	1 (1.9)	
Not evaluable	4 (3.8)	1 (1.9)	
Missing	6 (5.7)	5 (9.3)	

CI, confidence interval; CR, complete response; CRi, complete response with incomplete marrow recovery; ITT, intent‐to‐treat; nPR, nodular partial response; PR, partial response; PRL, partial response with lymphocytosis.

a Rate ratio and *P*‐values for ORR and ORR with PRL are based on Cochran–Mantel–Haenszel chi‐square test stratified by two randomization factors: refractory to purine analog therapy (yes or no) and del(17p) (yes or no).

Lymph node response was evaluated as one of the sensitivity analyses for ORR. The proportion of patients with lymph node response was significantly higher in the ibrutinib arm (84.9%) than in the rituximab arm (20.4%); the rate ratio was 4.17 (95% CI: 2.45–7.12; *P *<* *0.0001; Fig. S4).

At data cutoff, 33 deaths were observed at any time on study; 17 (16.0%) patients in the ibrutinib arm and 16 (29.6%) patients in the rituximab arm. With a median follow‐up of 17.8 months (range, 0.1–26.1 months), an improvement in OS was observed in the ibrutinib arm compared with the rituximab arm (HR = 0.446; 95% CI: 0.221–0.900; *P *=* *0.0206; Fig. 1B). The estimated 24‐month OS rate was 79.8% (95% CI: 68.9–87.2%) in the ibrutinib arm and 57.6% (95% CI: 36.2–74.1%) in the rituximab arm. Of note, 20 (37.0%) patients in the rituximab arm crossed over to receive ibrutinib therapy after confirmed PD.

Ibrutinib behaved as expected based on previous PK studies 24, 36. A summary of ibrutinib PK parameters in Chinese patients is presented in Table S3.

### Safety

At data cutoff, the median duration of treatment for the ibrutinib arm was nearly four times as long as the rituximab arm (16.4 vs. 4.6 months, respectively); the incidence of AEs was not adjusted for exposure. All‐grade AEs were comparable between both arms; grade ≥3 AEs were reported for 82.7% of patients in the ibrutinib arm and 59.6% of patients in the rituximab arm (Table 4). The most common all‐grade AEs in the ibrutinib arm were diarrhea, platelet count decreased, neutrophil count decreased, and cough. In the rituximab arm, the most common all‐grade AEs were neutrophil count decreased, platelet count decreased, and pyrexia (Table 4).

**Table 4:** Adverse events (safety population)

	Ibrutinib (*n* = 104)		Rituximab (*n* = 52)	
	All Grade	Grade ≥3	All Grade	Grade ≥3
AEs	103 (99.0)	86 (82.7)	47 (90.4)	31 (59.6)
Study drug‐related	95 (91.3)		36 (69.2)	
Leading to treatment discontinuation	13 (12.5)		4 (7.7)	
With outcome of death	9 (8.7)		3 (5.8)	
Serious AEs	45 (43.3)	41 (39.4)	17 (32.7)	16 (30.8)
Study drug‐related	25 (24.0)		10 (19.2)	
AEs occurring in ≥10% of patients				
Diarrhea	35 (33.7)	4 (3.8)	3 (5.8)	0
Platelet count decreased	31 (29.8)	8 (7.7)	15 (28.8)	3 (5.8)
Neutrophil count decreased	28 (26.9)	19 (18.3)	21 (40.4)	13 (25.0)
Cough	26 (25.0)	1 (1.0)	4 (7.7)	0
Pyrexia	25 (24.0)	1 (1.0)	14 (26.9)	1 (1.9)
Neutropenia	24 (23.1)	17 (16.3)	11 (21.2)	10 (19.2)
Rash	24 (23.1)	0	3 (5.8)	0
Upper respiratory tract infection	23 (22.1)	7 (6.7)	6 (11.5)	1 (1.9)
Lung infection	21 (20.2)	17 (16.3)	6 (11.5)	5 (9.6)
Fatigue	20 (19.2)	0	6 (11.5)	0
Thrombocytopenia	17 (16.3)	5 (4.8)	3 (5.8)	0
Anemia	16 (15.4)	2 (1.9)	5 (9.6)	0
Hemoglobin decreased	15 (14.4)	0	6 (11.5)	0
Nasopharyngitis	15 (14.4)	0	0	0
Nausea	15 (14.4)	0	1 (1.9)	0
Constipation	13 (12.5)	0	0	0
Lymphocyte count increased	13 (12.5)	11 (10.6)	0	0
Leukocytosis	12 (11.5)	12 (11.5)	0	0
Mouth ulceration	12 (11.5)	0	2 (3.8)	0
Vertigo	11 (10.6)	0	0	0
White blood cell count decreased	6 (5.8)	2 (1.9)	9 (17.3)	3 (5.8)
Chills	1 (1.0)	0	9 (17.3)	0

AE, adverse event.

Patients with multiple severity ratings for a given AE were counted only once under the maximum toxicity grade. Patients with missing toxicity grades are included in the all‐grade column.

The incidence of AEs leading to death was similar for both treatments; nine (8.7%) patients in the ibrutinib arm and three (5.8%) patients in the rituximab arm. With ibrutinib, death due to unknown cause and pneumonia was reported in two (1.9%) patients each. Two ibrutinib‐treated patients had multiple concurrent AEs, none of which could be excluded as cause of death (one patient died of femoral neck fracture, hemolytic anemia, staphylococcal sepsis, and hematoma; one patient died of pneumonia, respiratory failure, sepsis, septic shock, and Richter's transformation to diffuse large B‐cell lymphoma). The other AEs leading to death in the ibrutinib arm were single occurrences: atrial fibrillation, cerebral infarction, ketoacidosis, and lung infection. The patient who died of atrial fibrillation had multiple confounding factors, including cardiac and respiratory failure. In the rituximab arm, lung infection, cerebral hemorrhage, and viral pneumonia were the causes of the three deaths.

The incidence of bleeding AEs was 28.8% in the ibrutinib arm and 3.8% in the rituximab arm; most of the events were grade 1–2 in severity. Major hemorrhage events occurred in three (2.9%) patients in the ibrutinib arm and one (1.9%) patient in the rituximab arm. All major hemorrhage events were reported as serious AEs. Two bleeding events were fatal (hematoma due to femoral neck fracture in the ibrutinib arm and cerebral hemorrhage in the rituximab arm). Patients who experienced major hemorrhage while on ibrutinib were taking concomitant aspirin at the time they entered the study until the AE was reported.

Infections were reported in 68.3% of patients in the ibrutinib arm and 40.4% of patients in the rituximab arm. Upper respiratory tract infections (22.1% in the ibrutinib arm and 11.5% in the rituximab arm) and unspecified lung infections (20.2% in the ibrutinib arm and 11.5% in the rituximab arm) were the most frequently reported in both treatment arms (Table 4).

The incidence of common cytopenic events (neutropenia, thrombocytopenia, anemia, and febrile neutropenia) was similar for both the ibrutinib and rituximab arms (75.0% and 71.2%, respectively). Incidence of grade 3 or 4 neutropenia (combined neutropenia and neutrophil count decreased), thrombocytopenia (combined thrombocytopenia and platelet count decreased), anemia (combined anemia and hemoglobin decreased), and febrile neutropenia were 33.7%, 12.5%, 1.9%, and 2.9%, respectively, in the ibrutinib arm and 44.2%, 5.8%, 0%, and 1.9%, respectively, in the rituximab arm.

Atrial fibrillation was only reported in the ibrutinib arm; six (5.8%) patients reported atrial fibrillation, which was the most frequently reported cardiac event. One patient experienced atrial fibrillation with cardiac and respiratory failure occurring at the same time, leading to death.

Adverse events of hypertension were the most frequently reported vascular disorders event (5.8% each for the ibrutinib and rituximab arms). Grade 3 or 4 events were reported in 1.9% of patients in the ibrutinib arm and 1.9% of patients in the rituximab arm.

Rashes of all grades were reported in 24.0% of patients in the ibrutinib arm and 7.7% of patients in the rituximab arm. All rashes were grade 1 or 2 in severity, with the exception of a grade 3 case of maculopapular rash reported by a patient in the ibrutinib arm. Eye disorder AEs observed in the ibrutinib arm (7.7%) and in the rituximab arm (1.9%) were mostly grade 1 in severity.

## Discussion

In this randomized, open‐label, phase 3 study, ibrutinib significantly improved PFS, ORR, and OS compared with rituximab in patients from the Asia‐Pacific region with R/R CLL/SLL. Although rituximab monotherapy is not often used today for R/R CLL/SLL, it was a reasonable choice as a comparator based on the limited treatment options available in the region at the time this study was initiated. It is also acknowledged that there are various different rituximab monotherapy doses and schedules which could be used. The study did not utilize the most dose‐dense schedule available but did use a schedule consistent with the comparator in another global study of a B‐cell signaling inhibitor 28.

Differences in baseline characteristics were observed between the treatment arms. A higher percentage of patients in the rituximab arm had ≥3 prior therapies and prior purine analog therapy. The rituximab arm also had a higher percentage of patients with bulky disease; the difference in the proportion of bulky disease in both arms was random. However, multivariate Cox regression analysis of PFS showed that these differences did not influence treatment outcomes. Ibrutinib showed favorable efficacy in this population of patients with advanced disease and a high proportion of poor prognostic factors.

The PFS advantage with ibrutinib (>80% reduction in risk of PD or death) is similar to that previously reported for the global study of ibrutinib versus ofatumumab, another anti‐CD20 antibody, in patients with previously treated CLL 26. The median PFS with rituximab was longer in our study than previously reported for a similar population 28. This difference may be due to a lower rate of previous rituximab treatment in our study, as rituximab therapy is not available to all patients in China.

The investigator‐assessed ORR in this study is lower than that reported in prior trials with single‐agent ibrutinib (~70%) 25, 26. Because previous studies have shown that the quality of responses improves with longer ibrutinib treatment 37, 38, the ORR of ibrutinib is expected to result in continued improvement with longer follow‐up. Consistent with this, the ORR in the ibrutinib arm from the updated analysis (16.4‐month median duration of exposure) was higher (53.8%) than that observed at the interim analysis (45.3%; 12.6‐month median duration of exposure). In addition, ORR including PRL was 67.9% in the ibrutinib arm, indicating that there is further potential for the ORR to increase. Lymph node response was also greater in the ibrutinib arm than in the rituximab arm.

The rituximab ORR (7.4%) observed in this study is in line with reported rates of 6–35% using standard dosing regimens 39, 40, 41, 42. In the Furman et al. study of idelalisib and rituximab, an ORR of 13% was observed with rituximab in relapsed CLL. Because of the different populations used in the Furman et al. study and our study (response evaluable vs. ITT population, respectively), no comparisons can be made.

Overall survival was significantly improved in the ibrutinib arm compared with the rituximab arm despite the inclusion of 20 (37.0%) rituximab patients who crossed over to receive ibrutinib. The median OS was not reached for the ibrutinib arm and was 26.1 months (95% CI: 17.5–26.1) for the rituximab arm.

Ibrutinib displayed a manageable safety profile which was consistent with results from earlier studies 25, 26, 43. The frequency of treatment discontinuations due to AEs (12.5%) and dose reductions (4.8%) was low. It should be noted that the median duration of treatment was approximately four times longer for ibrutinib than rituximab, and the incidence of AEs was not adjusted for exposure.

Atrial fibrillation occurred only in the ibrutinib arm (5.8%), and affected patients had comorbidities including hypertension, cardiac conditions, or diabetes. One of eight patients with a history of atrial fibrillation or abnormal heart rhythm developed atrial fibrillation while on the study. In a retrospective database study, 6.1% of patients had a history of atrial fibrillation at the time of CLL diagnosis, and 6.1% of patients without a history of atrial fibrillation at diagnosis had atrial fibrillation during follow‐up 44, which is comparable to the incidence observed with ibrutinib in this study. This finding supports the use of ibrutinib in patients with a history of atrial fibrillation or other pre‐existing risk factors with careful monitoring and dose adjustments.

The incidence of cytopenic events of any grade was comparable between both treatment groups. Grade 3 or 4 neutropenia was more frequent with rituximab, and grade 3 or 4 thrombocytopenia was more common with ibrutinib. Most of the neutropenia and thrombocytopenia events occurred early in treatment and were manageable with colony stimulating factors or transfusions. Cytopenias are common manifestations of CLL, and clinical laboratory results showed improvements in mean and median platelet and hemoglobin counts over time in both arms.

In conclusion, this is the first study of ibrutinib in a predominantly Asian population of patients with CLL/SLL and the first study to compare ibrutinib with rituximab. Ibrutinib significantly improved PFS, ORR, and OS compared with rituximab; the results were robust and internally consistent. Ibrutinib displayed a manageable safety profile with no new or unexpected events reported. The findings in this study are consistent with those reported in previous studies of single‐agent ibrutinib 25, 26, 43, indicating that the efficacy and safety profiles of ibrutinib in Asian patients are comparable to those observed in the general patient population. Together, these data demonstrate the favorable benefit‐risk profile of ibrutinib for the treatment of R/R CLL/SLL in high‐risk patients from the Asia‐Pacific region.

## Conflict of Interest

M.S., S.S., A.H., and Jingzhao W. are current employees, and P.W. is a former employee of Janssen Research & Development, LLC and declare that, except for income received from their primary employer, no financial support or compensation has been received from any individual or corporate entity over the past three years for research or professional service that could be perceived as constituting a potential conflict of interest. M.S., S.S., and A.H. are shareholders of Johnson & Johnson, the parent company of the Janssen companies. L.Q. has received speaker bureau fees from Xi'an Janssen Pharmaceutical Ltd., Roche, and Celgene. X.J., J.J., D.Z., X.C., M.H., J.H., Y.H., X.K., Junmin L., Y. Liang, T.L., Y. Lv, H.R., A.S., Jianmin W., C.Z., and Jianyong L. do not have any potential conflicts of interest to disclose.

## Supporting information


**Appendix S1**. Additional inclusion and exclusion criteria, criteria for disease progression, and criteria for response categories.
**Figure S1**. Diagram of the study design prior to implementation of crossover.
**Figure S2**. Diagram of the study design after implementation of crossover.
**Figure S3**. Patient disposition during the treatment phase.
**Figure S4.** Change in the measured size of lymph nodes from baseline (ITT population).
**Table S1.** IWCLL 2008 response criteria.^1,2^
**Table S2.** Summary of PFS, ORR, and OS at the interim analysis (ITT population).
**Table S3.** Summary of ibrutinib PK parameters.
