# Supplementary material for: Ibrutinib versus rituximab in relapsed or refractory chronic lymphocytic leukemia or small lymphocytic lymphoma: a randomized, open‐label phase 3 study
Source: Cancer Med. 2018 Mar 13;7(4):1043–55. doi: 10.1002/cam4.1337 (PMC5911578; doi:10.1002/cam4.1337)
Supplement: Supplementary file 1 — Appendix S1. Additional inclusion and exclusion criteria, criteria for disease progression, and criteria for response categories. Figure S1. Diagram of the study design prior to implementation of crossover. Figure S2. Diagram of the study design after implementation of crossover. Figure S3. Patient disposition during the treatment phase. Figure S4. Change in the measured size of lymph nodes from baseline (ITT population). Table S1. IWCLL 2008 response criteria.1,2 Table S2. Summary of PFS, ORR, and OS at the interim analysis (ITT population). Table S3. Summary of ibrutinib PK parameters. [file CAM4-7-1043-s001.docx]

**Supplementary Appendix**

**Table of Contents**

Additional Key Inclusion and Exclusion Criteria2

Supplementary Figure 1. Diagram of the Study Design Prior to Implementation of Crossover4

Supplementary Figure 2. Diagram of the Study Design After Implementation of Crossover5

Criteria for Defining Disease Progression6

Criteria for Response Categories6

Supplementary Table 1. IWCLL 2008 Criteria8

Supplementary Table 2. Summary of PFS, ORR, and OS at the Interim Analysis 10

Supplementary Figure 3. Patient Disposition During the Treatment Phase12

Supplementary Figure 4. Change in the Measured Size of Lymph Nodes from
Baseline (ITT Population)13

Pharmacokinetic Evaluations14

Supplementary Table 3. Summary of Ibrutinib PK Parameters14

References15

Additional Key Inclusion and Exclusion Criteria

Eligible patients also had Eastern Cooperative Oncology Group (ECOG) performance status score of 0 to 1; measurable nodal disease by computed tomography (CT); absolute neutrophil count ≥750 cells/µL that was independent of growth factor support 7 days prior to assessment; platelet count ≥30,000 cells/µL without transfusion support 7 days prior to assessment; serum aspartate transaminase or alanine transaminase <2.5 times the upper limit of normal (ULN); total bilirubin ≤1.5 times the ULN; estimated creatine clearance ≥30 mL/min; negative serum or urine pregnancy test (women); and agreed to use an effective form of contraception during the study.

Patients were not considered appropriate candidates for treatment or retreatment with purine analog-based therapy if they had at least 1 of the following criteria: (a) Failure to respond (stable disease or disease progression on treatment), or a progression-free interval of less than 24 months from first dose of treatment with a purine analog-based therapy after at least 2 cycles; (b) Age ≥70 years who had received 1 prior systemic therapy consisting of at least 2 cycles of chemotherapy or chemoimmunotherapy; (c) Age ≥65 years who had received ≥1 prior treatment including at least 2 cycles of an alkylating agent-based (or purine analog-based) chemotherapy regimen and had at least 1 comorbidity (estimated creatinine clearance <70 mL/min, platelet count <100,000/μL or hemoglobin <10 g/dL, history of clinically significant autoimmune cytopenia [autoimmune hemolytic anemia or immune thrombocytopenia], or ECOG performance score = 1); (d) History of purine analog-associated autoimmune anemia or autoimmune thrombocytopenia, or (e) Screening FISH showing 17p del is present and defined as positive per the assay specification (Abbott Vysis kit) either alone or in combination with other cytogenetic abnormalities, provided they have received at least 1 prior therapy.

Patients were ineligible if they had prior exposure to ibrutinib; were refractory to prior rituximab (relapsed within 6 months at the end of rituximab-based therapy); received chemotherapy, external beam radiation therapy, anticancer antibodies, or investigational drug within 30 days prior to first dose of study drug; received prior autologous transplant within 6 months prior to first dose of study drug; received prior stem cell transplant; had known central nervous system leukemia/lymphoma or Richter’s transformation; used corticosteroid (>20 mg) within 1 week prior to first dose of study drug; used required anticoagulation with warfarin or equivalent vitamin K antagonist; required treatment with strong CYP3A4/5 inhibitors; had a history of prior malignancy (except certain skin cancers), active clinically significant cardiovascular disease, or uncontrolled active system infections; and had a history of human immunodeficiency virus or active hepatitis B or C infection.

**Supplementary Figure 1. Diagram of the Study Design Prior to Implementation of Crossover**

**
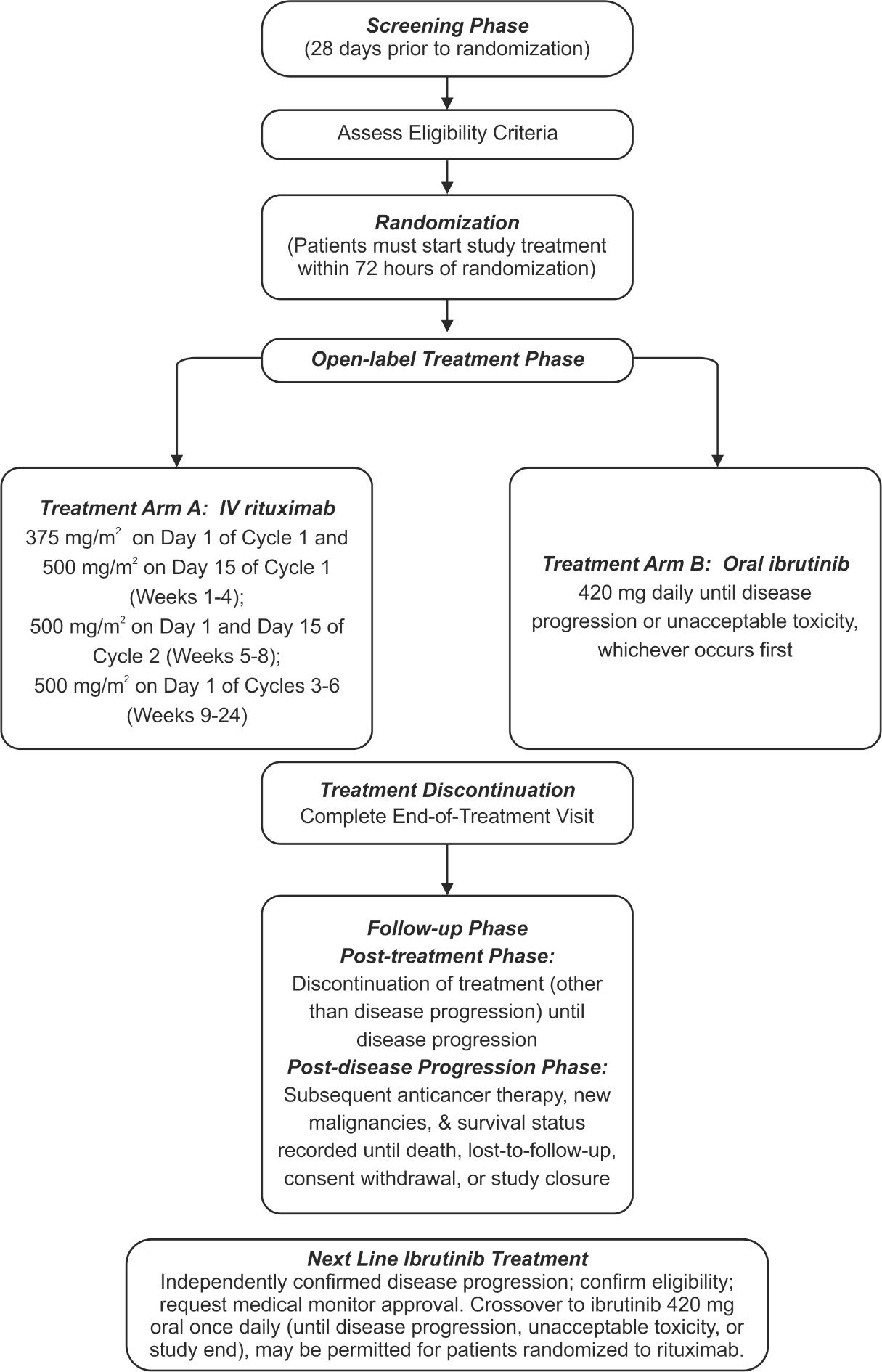
**

**Supplementary Figure 2. Diagram of the Study Design After Implementation of Crossover**


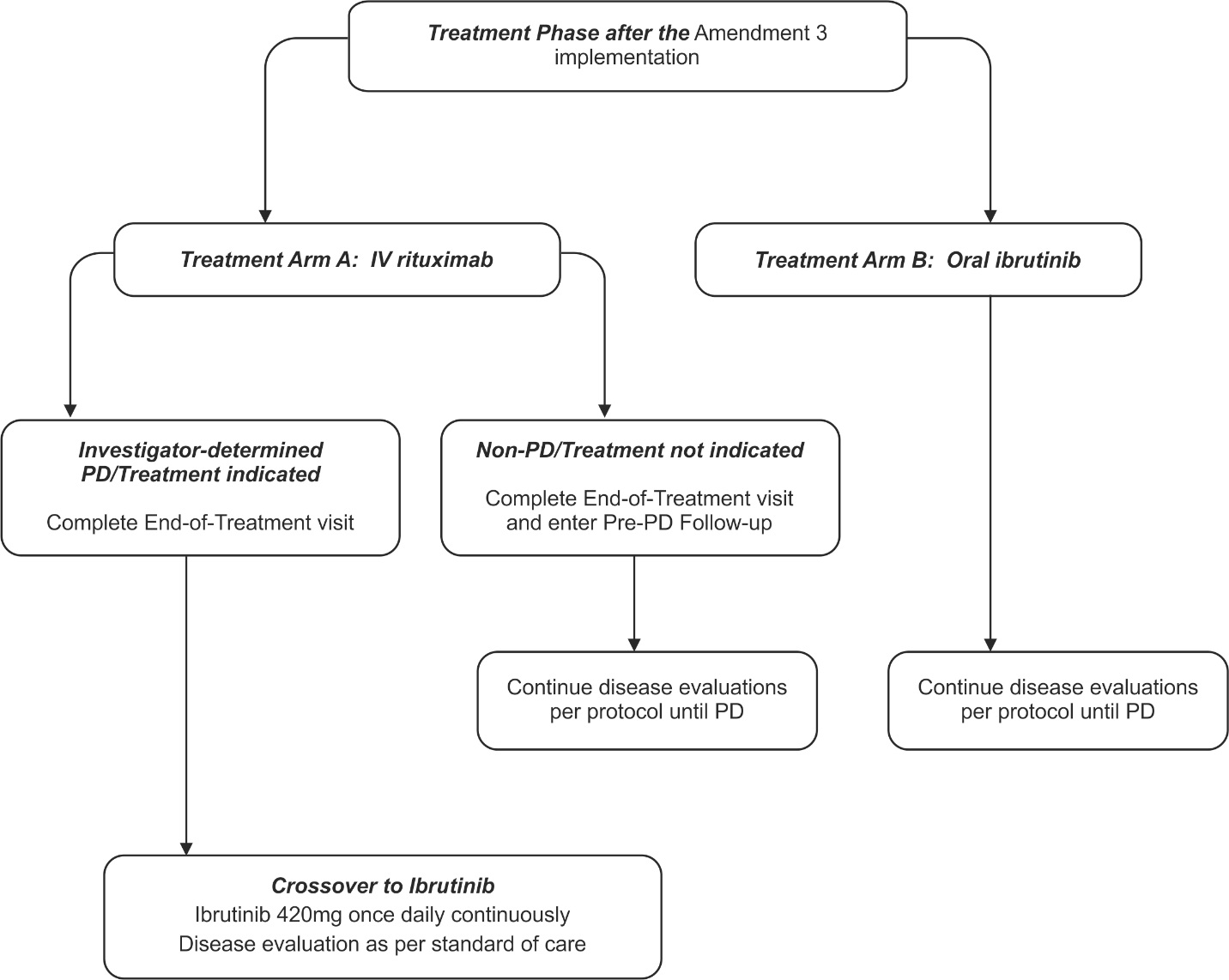


Criteria for Defining Disease Progression

A CT scan was required to evaluate all cases of suspected progressive disease regardless of the modality of disease progression (e.g. lymph node, lymphocytosis, or

transformation). Progressive disease required at least 1 of the following: (1) new enlarged nodes >1.5 cm, new hepatomegaly or splenomegaly, or other organ infiltrates; (2) ≥50% increase from nadir in existing lymph node (must reach >1.5 cm in the longest

diameter) or ≥50% increase from nadir in sum of product of diameters of multiple nodes; (3) ≥50% increase from nadir in enlargement of liver or spleen; (4) ≥50% increase from baseline in lymphocyte count (and to ≥5 x10^9^/L) unless considered

treatment-related lymphocytosis; (5) new cytopenia (hemoglobin [Hgb] or platelets) attributable to CLL; or (6) transformation to a more aggressive histology (e.g., Richter’s Transformation).

Criteria for Response Categories

Objective responses for both treatment arms were categorized based on the International Workshop on Chronic Lymphocytic Leukemia (IWCLL) 2008 criteria (Supplementary Table 1) and the definitions listed below.

A complete response (CR) was defined by the presence of all of the following: (1) no significant lymphadenopathy (>1.5 cm) palpable on examination or by CT; (2) no hepatosplenomegaly on examination or by CT; (3) no constitutional symptoms (i.e., no fever >38°C for ≥2 weeks, no unintentional ≥10% body weight loss within 6 months, no night sweats for >1 month without other evidence of infection, no fatigue interfering with work or usual activities); (4) neutrophils >1.5 x 10^9^/L, platelets >100 x 10^9^/L, and
Hgb >11 g/dL without recent growth factor or transfusions; and (5) absolute lymphocyte count <4,000/μL. Marrow aspirate and biopsy were performed after all other criteria met the definition of CR. To define a CR, the marrow sample was at least normocellular for age, with less than 30% of nucleated cells being lymphocytes. B-lymphoid nodules were absent and morphologic review of marrow biopsy revealed no clonal infiltrate.

A CR with an incomplete marrow recovery (CRi) was defined as a CR with an

incomplete recovery of the patient’s bone marrow. Patients who had a CRi fulfilled all criteria for a CR, but continued to have persistent anemia, thrombocytopenia, or neutropenia. These cytopenias were due to drug toxicity in the bone marrow and were not due to any evidence of CLL. If the marrow was hypocellular, a repeat determination was performed after 4 weeks, or when peripheral blood counts recovered. However, this time interval did not exceed 6 months.

A nodular partial response (nPR) was a response where patients met the criteria for a CR, but the bone marrow biopsy showed that B-lymphoid nodules were still present, which may have represented a clonal infiltrate. These nodules were residual disease and therefore the patient was termed an nPR.

A partial response (PR) was a response with ≥50% drop in lymphocyte count from baseline or ≤4.0 x 10^9^/L and all of the following: (1) ≥50% decrease in the sum products of up to 6 lymph nodes, a ≥50% decrease in the longest diameter of the single lymph node, or normalization of lymphadenopathy when compared with baseline; (2) no new enlarged lymph nodes by physical examination or CT AND no increase in any

lymph node by CT; and (3) when abnormal, a ≥50% decrease in the enlargement of the spleen/liver from baseline or normalization by CT. In addition, a response in at least 1 of the following evaluable criteria, independent of growth factor support or transfusion was needed: (1) neutrophils >1.5 x10^9^/L, or ≥50% improvement over baseline; (2) platelets >100,000/μL or ≥50% improvement over baseline; and (3) Hgb >11 g/dL or ≥50% improvement over baseline.

PR with lymphocytosis (PRL) was a response where the patient achieved all PR criteria with the exception of persistent lymphocytosis.

Stable disease (SD) was defined as not meeting the criteria for CR, Cri, nPR, PRL, or progressive disease.

**Supplementary Table 1. IWCLL 2008 Response Criteria**^1,2^

| **Parameter** | **Complete Response (CR)** | **Partial Response (PR)** | **Progressive Disease** |
| --- | --- | --- | --- |
| **Group A** | | | |
| Lymphadenopathy^a^ | None >1.5cm | Decrease ≥50%^b^ | Increase ≥50% |
| Hepatomegaly | None | Decrease ≥50% | Increase ≥50% or appearance of new hepatomegaly |
| Splenomegaly | None | Decrease ≥50% | Increase ≥50% or appearance of new splenomegaly |
| Blood lymphocytes | <4000/μL | Decrease ≥50% from baseline | Increase ≥50% over baseline^d^ |
| Marrow^c^ | Normocellular, <30% lymphocytes, no B lymphoid nodules. Hypocellular marrow defines CRi |  |  |
| **Group B** | | | |
| Platelet count | >100,000/μL | >100,000/μL or increase ≥50% over baseline | Decrease of ≥50% from baseline secondary to CLL |
| Hemoglobin | >11 g/dL | >11g/dL or increase ≥50% over baseline | Decrease of >2g/dL from baseline secondary to CLL |
| Neutrophils^c^ | >1500/μL | >1500/μL or increase ≥50% over baseline | N/A |

Note: Group A defined the tumor load and Group B defined the function of the hematopoietic system.

^a^Sum of the products of multiple lymph nodes (as evaluated by CT scans) or the longest diameter of 1 target lymph node.

^b^Defined as a decrease in lymph nodes of ≥50% either in the sum products of the diameter of up to 6 lymph nodes, or in the largest diameter of the enlarged lymph node detected prior to the therapy, as well as no increase in any lymph node and no new enlarged lymph nodes. Note: in small lymph nodes <2 cm, an increase of <25% was not considered to be significant.

^c^This parameter was not relevant for the progressive disease category unless confirming cytopenic progression.

^d^Patients with treatment-related lymphocytosis remained on study treatment in the absence of other criteria for progressive disease.

CR: All of the criteria needed to be met and patients had to lack disease-related constitutional symptoms. Bone marrow aspirate required to confirm CR.

PR: All abnormal criteria were met from Group A plus 1 of the criteria from Group B was met. Note: If all PR criteria with the exception of absolute lymphocyte count were met, this was consistent with a PR with lymphocytosis. If only 1 measurable Group A criterion was present at baseline (e.g., enlarged lymph nodes but no other abnormality), per recent clarification of the IWCLL criteria,^2^ these patients were still considered evaluable for PR if the given parameter improved by at least 50% for a minimum of 2 months. Patients were also required to have 1 Group B parameter, which could either be improvement in a previously abnormal finding or the persistence of a normal value for at least 2 months as a result of therapy.

SD: The absence of progressive disease and the failure to achieve a CR, CRi, nPR, PR, or PR with lymphocytosis

Progressive Disease: At least 1 of the above criteria from Group A or B were met; or transformation to more aggressive histology (e.g., Richter’s transformation).

**Supplementary Table 2. Summary of PFS, ORR, and OS at the Interim Analysis (ITT Population)**

| **Endpoint** | **Ibrutinib**  **(n=106)** | **Rituximab**  **(n=54)** | **Ibrutinib vs. Rituximab** |
| --- | --- | --- | --- |
| **PFS** |  |  |  |
| Median, months (95% CI) | 18.5 (18.3-NE) | 8.5 (8.3-10.1) | – |
| 12-month PFS rate (95% CI) | 0.815 (0.714-0.883) | 0.247 (0.117-0.403) | – |
| Hazard ratio (95% CI) | – | – | 0.197 (0.107-0.364) |
| *p*-value | – | – | <0.0001 |
| **ORR, n (%)** |  |  |  |
| CR, CRi, nPR, and PR | 48 (45.3) | 3 (5.6) | – |
| Rate ratio (95% CI) | – | – | 8.24 (2.68-25.34) |
| *p*-value | – | – | <0.0001 |
| CR, CRi, nPR, PR, and PRL | 60 (56.6) | 3 (5.6) | – |
| Rate ratio (95% CI) | – | – | 10.29 (3.36-31.49) |
| *p*-value | – | – | <0.0001 |
| Best overall response |  |  |  |
| CR | 2 (1.9) | 0 | – |
| PR | 46 (43.4) | 3 (5.6) | – |
| PRL | 12 (11.3) | 0 | – |
| **OS** |  |  |  |
| Median, months (95% CI) | NE | 19.5 (16.9-NE) | – |
| 12-month survival rate (95% CI) | 0.900 (0.815-0.947) | 0.851 (0.692-0.931) | – |
| Hazard ratio (95% CI) | – | – | 0.453 (0.183-1.124) |
| *p*-value | – | – | 0.0800 |

CI = confidence interval; CR = complete response; CRi =complete response with incomplete marrow recovery; ITT = intent-to-treat; nPR = nodular partial response; NE = not evaluable; PFS = progression-free survival; PR = partial response; PRL = partial response with lymphocytosis; ORR = overall response rate; OS = overall survival.

**Supplementary Figure 3. Patient Disposition During the Treatment Phase**

**
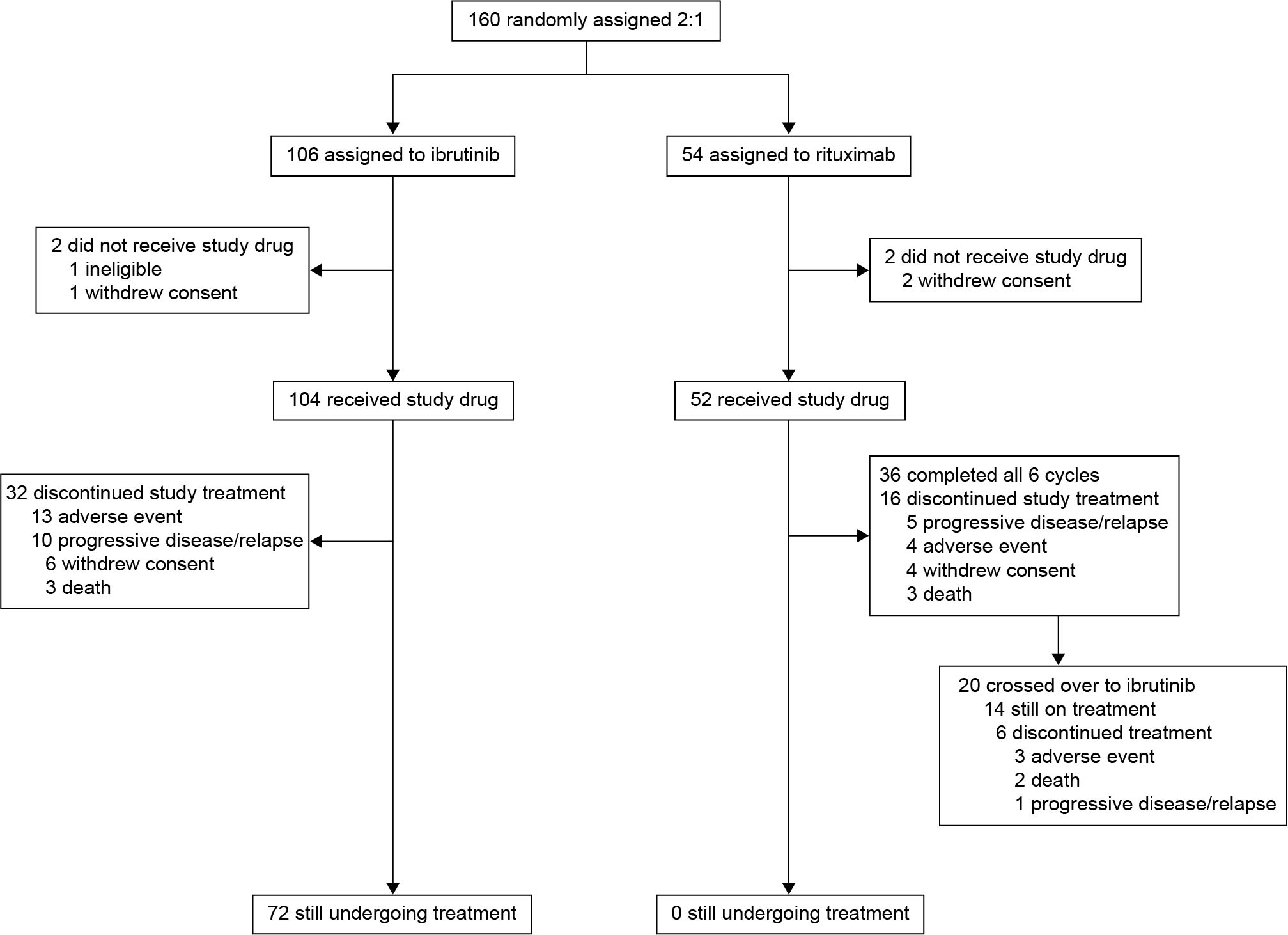
**

**Supplementary Figure 4. Change in the Measured Size of Lymph Nodes from Baseline (ITT Population).** The greatest percentage change in the sum of the products of the perpendicular diameters of measured lymph nodes for each patient from baseline are shown for the ibrutinib arm (blue) and rituximab arm (red).

**
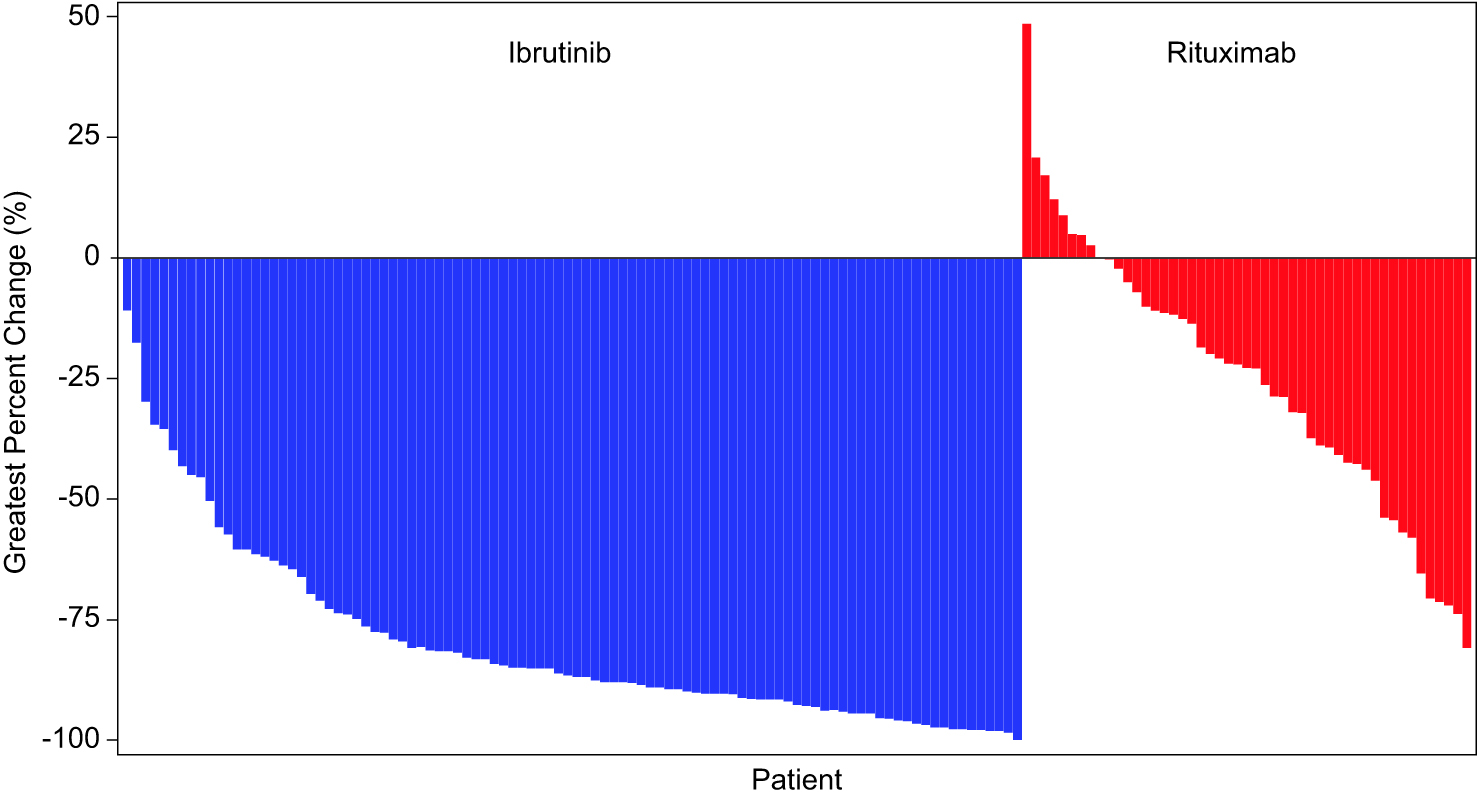
**

Pharmacokinetic Evaluations

Pharmacokinetic samples were collected from a subset of patients (n=20) randomized to receive ibrutinib. Ten blood samples were collected per patient at the week 1 and week 4 visits prior to ibrutinib intake and at the following time points after drug intake: 30 minutes, and 1, 2, 3, 4, 6, 8, 10, and 24 hours. Plasma samples were analyzed by a validated and specific liquid chromatography tandem mass spectrometry (LC-MS/MS) method for the determination of ibrutinib and its metabolite PCI-45227. Pharmacokinetic parameters were derived from plasma concentration versus time data for ibrutinib.

**Supplementary Table 3. Summary of Ibrutinib PK Parameters**

| **PK Parameter, mean (SD)** | **Single Dose Phase (N=20)** | **Multiple Dose Phase (N=20)** |
| --- | --- | --- |
| t_max_, h | 2.00 (0.98-3.00)^a,e^ | 2.00 (0.50-3.03)^a,e^ |
| C_min_, ng/mL | NR | 3.73 (7.80)^e^ |
| C_max_, ng/mL | 107 (87.7)^e^ | 126 (127)^e^ |
| t_1/2,λ_, h | 4.83 (2.72)^b^ | 7.34 (3.43)^b^ |
| AUC_0-24_, ng·h/mL | 439 (331)^d^ | 515 (737)^d^ |
| Accumulation index | NR | 1.15 (0.23-10.8)^a,c,f^ |

AUC_0-24_ = area under the plasma concentration-time curve from time 0 to 24 hr; C_max_ = maximum observed plasma concentration; C_min_ = minimum observed plasma concentration; NR = not reportable; PK = pharmacokinetic; t_1/2, λ_ = elimination half-life associated with the terminal slope (λ_z_) of the semi-logarithmic drug concentration-time curve, calculated as 0.693/λ_z_; t_max_ = time to reach maximum plasma concentration.

^a^Median (range)

^b^n=13

^c^n=17

^d^n=18

^e^n=19

^f^Median (range) reported due to large interpatient variability.

**References**

1. Hallek M, Cheson BD, Catovsky D, Caligaris-Cappio F, Dighiero G, Dohner H, Hillmen P, Keating MJ, Montserrat E, Rai KR, Kipps TJ, International Workshop on Chronic Lymphocytic Leukmia. Guidelines for the diagnosis and treatment of chronic lymphocytic leukemia: a report from the International Workshop on Chronic Lymphocytic Leukemia updating the National Cancer Institute-Working Group 1996 guidelines. Blood 2008; 111: 5446-5456

2. Hallek M, Chenson BD, Catovsky D, Caligaris-Cappio F, Dighiero G, Doehner H, Hillmen P, Keating M, Montserrat E, Rai KR, Kipps TJ. Response assessment in chronic lymphocytic leukemia treated with novel agents causing an increase of peripheral blood lymphocytes. Blood 2012. http://www.bloodjournal.org/content/111/12/5446.e-letters
